# Insulin resistance in type 1 diabetes is a key modulator of platelet hyperreactivity

**DOI:** 10.1007/s00125-025-06429-z

**Published:** 2025-04-30

**Authors:** Rebecca C. Sagar, Daisie M. Yates, Sam M. Pearson, Noppadol Kietsiriroje, Matthew S. Hindle, Lih T. Cheah, Beth A. Webb, Ramzi A. Ajjan, Khalid M. Naseem

**Affiliations:** 1 Leeds Institute of Cardiovascular and Metabolic Medicine, University of Leeds, Leeds, UK; 2 Leeds Teaching Hospitals Trust, Leeds, UK; 3 Endocrinology and Metabolism Unit, Faculty of Medicine, Prince of Songkla University, Hatyai, Thailand; 4 Centre for Biomedical Science Research, School of Health, Leeds Beckett University, Leeds, UK

**Keywords:** Estimated glucose disposal rate, Inhibition, Insulin resistance, Integrin α_IIb_β_3_, Phosphatidylserine, Platelets, P-selectin, Type 1 diabetes

## Abstract

**Aims/hypothesis:**

Individuals with type 1 diabetes are at increased cardiovascular risk, particularly in the presence of insulin resistance. A prothrombotic environment is believed to contribute to this risk but thrombotic pathways in type 1 diabetes are only partially understood and the role of platelets is incompletely studied. We hypothesised that platelets from individuals with type 1 diabetes exhibit platelet hyperactivity due to both increased propensity for activation and diminished sensitivity to inhibition, with an amplified maladaptive phenotype in those with insulin resistance.

**Methods:**

Blood samples were obtained from individuals with type 1 diabetes enrolled on the ‘Double diabEtes and adVErse cLinical Outcome: identification of mechanistic Pathways’ (DEVELOP) study with insulin resistance assessed as estimated glucose disposal rate (eGDR), whereby eGDR >8 or <6 mg kg^−1^ min^−1^ indicates normal insulin sensitivity or advanced insulin resistance, respectively. Platelet function was analysed using whole blood multiparameter flow cytometry to simultaneously measure three distinct markers of activation, including integrin α_IIb_β_3_ (PAC-1 binding), P-selectin (CD62P) and phosphatidylserine (PS) (Annexin V). Both activation and inhibition responses of the platelets were investigated, which were subjected to the machine learning tool Full Annotation Shape-constrained Trees (FAUST) to characterise platelet subpopulations.

**Results:**

A total of 32 individuals with type 1 diabetes were studied (median age [range] of 24 [18–34] years, 59% male, diabetes duration [mean ± SD] of 14.0 ± 6.3 years and HbA_1c_ of 65.3 ± 14.0 mmol/mol [8.1%]). An increased basal expression, measured as mean fluorescence intensity, of all three platelet activation markers was detected in the type 1 diabetes group compared with healthy control participants (CD62P expression 521 ± 246 vs 335 ± 67; *p*<0.001, PAC-1 370 ± 165 vs 231 ± 88; *p*=0.011 and PS 869 ± 762 vs 294 ± 109; *p*=0.001). Following platelet stimulation, an enhanced activation of these markers was found in the type 1 diabetes group. Within the type 1 diabetes group, those with advanced insulin resistance (eGDR<6 mg kg^−1^ min^−1^) showed increased platelet activation compared with individuals with normal insulin sensitivity (eGDR>8 mg kg^−1^ min^−1^) with single agonist stimulation CD62P expression (29,167 ± 2177 vs 22,829 ± 2535, *p*<0.001 and PAC-1 19,339 ± 11,749 and 5187 ± 2872, *p*=0.02). Moreover, individuals with type 1 diabetes showed reduced sensitivity to platelet inhibition by prostacyclin (PGI_2_) compared with control participants. Stratification of individuals with type 1 diabetes by insulin resistance demonstrated that in the presence of PGI_2_, suppression of stimulated CD62P was 17 ± 11% and 33 ± 12% (*p*=0.02) for advanced insulin resistance and normal insulin sensitivity groups, respectively, with even larger differences demonstrated for PAC-1 (48 ± 17% and 75 ± 7%; *p*=0.006) and PS exposure (33 ± 12% and 84 ± 10%; *p*=0.001). Furthermore, FAUST analysis showed that, under basal conditions, there was a different distribution of the eight platelet subpopulations comparing advanced insulin resistance and normal insulin sensitivity groups, with differences also detected following PGI_2_ inhibition.

**Conclusions/interpretation:**

Our novel characterisation of platelets in type 1 diabetes shows a maladaptive phenotype with increased basal activity together with hyperactivation following stimulation and diminished responses to inhibition. Insulin resistance appears to further drive this adverse thrombotic phenotype, suggesting an enhanced platelet-driven cardiovascular risk in those with type 1 diabetes and reduced insulin sensitivity.

**Graphical Abstract:**

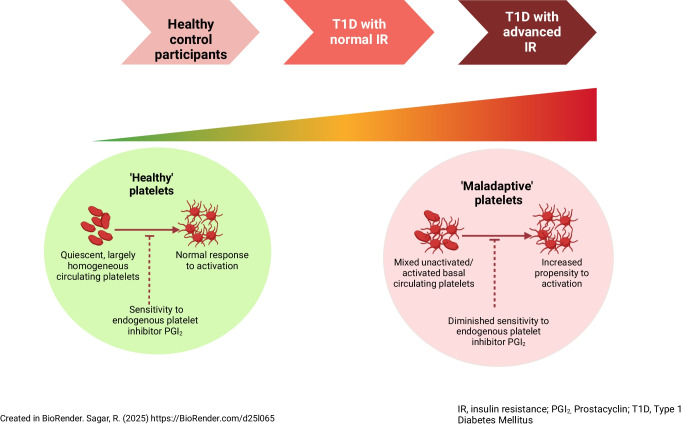

**Supplementary Information:**

The online version contains peer-reviewed but unedited supplementary material available at 10.1007/s00125-025-06429-z.


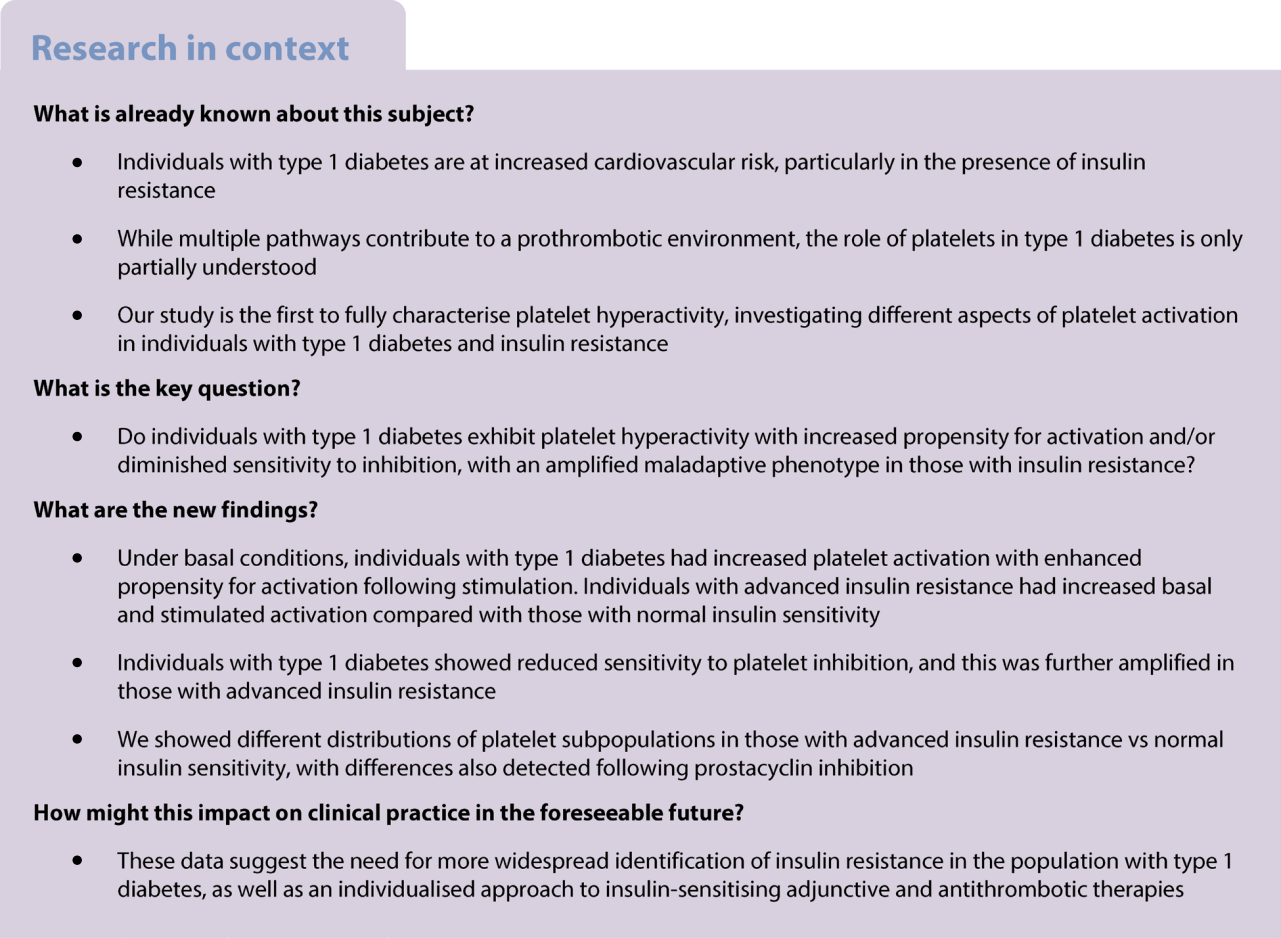


## Introduction

Diabetes is associated with a prothrombotic state that contributes to premature cardiovascular morbidity and mortality [1–3]. Elevated thrombotic risk in individuals with diabetes is driven by both a hypercoagulable state and increased platelet reactivity, although to date studies have largely focused on individuals with type 2 diabetes [4]. The evidence supporting platelet hyperactivity in individuals with type 1 diabetes is largely limited to data from early aggregation studies ([5–7] and evidence of elevated expression of surface P-selectin, as well as increased circulating levels of platelet–monocyte aggregates [8]. In addition to platelet stimulants, in vivo platelet reactivity is restricted by their exposure to endothelial-derived inhibitors prostacyclin (PGI_2_) and nitric oxide (NO). Therefore, changes to vascular function, sensitivity to these inhibitors or platelet activators are equally likely to alter thrombotic risk [9, 10]. Early work suggested impaired platelet response to inhibitors in individuals with type 1 diabetes and type 2 diabetes compared with healthy control groups [11], with subsequent work showing reduced sensitivity of type 2 diabetes platelets to PGI_2_ [12, 13] together with hyposensitivity to NO [14]. This implicates failure of platelet suppression as a key mechanism for platelet hyperactivity in type 2 diabetes and it remains unclear whether this applies to individuals with type 1 diabetes.

While hyperglycaemia is generally believed to modulate platelet function in type 1 diabetes [15, 16], the role of other metabolic factors, including insulin resistance, remains unclear. Importantly, insulin resistance in individuals with type 1 diabetes is associated with adverse vascular outcomes by mechanisms that are not fully understood [17–19]. In over 17,000 individuals with type 1 diabetes, Nyström et al have shown that insulin resistance, measured by estimated glucose disposal rate (eGDR) [18], predicted cardiovascular morbidity and mortality independently of blood glucose levels, measured as HbA_1c_ [20]. Similar findings have been documented by others, demonstrating a link between insulin resistance and vascular complications in individuals with type 1 diabetes [19–21].

Given these observations and the established role of platelets in cardiovascular pathology, we hypothesised that platelets from young individuals with type 1 diabetes exhibit platelet hyperactivity due to both increased propensity for activation and diminished sensitivity for inhibition, with this maladaptive phenotype correlating with severity of insulin resistance. A key advantage of studying the type 1 diabetes population is the limited number of confounders compared with type 2 diabetes, and therefore it is easier to disentangle the roles of glycaemia and insulin resistance in platelet dysfunction. The current study investigated multiple aspects of platelet activation and inhibition using a multiparameter flow cytometry panel in a younger cohort of type 1 diabetes with no advanced complications and receiving only glucose-lowering therapies.

Our overall aim was to comprehensively analyse platelet function in this cohort, with special focus on the role of insulin resistance through three inter-linked objectives: (1) understand the susceptibility of platelets to activation; (2) investigate the potential differential platelet response to inhibition; and (3) analyse the effects of insulin resistance on platelet activation and/or sensitivity to inhibition.

## Methods

### Study design and participants

Samples from participants with type 1 diabetes were obtained from those participating in the ‘Double diabEtes and adVErse cLinical Outcome: identification of mechanistic Pathways’ (DEVELOP) study. Ethical approval was granted by the Health Research Authority (Research Ethics Committee reference: 19/NE/0349, Integrated Research Application System number 259072).

Inclusion criteria were: (1) confirmed diagnosis of type 1 diabetes (supported by clinical history, positive islet autoantibodies and/or low C-peptide levels) for a minimum of 3 years and on current treatment with insulin (injections or insulin pump); (2) aged 18 years or older at the time of study visit and under 40 years old; (3) BMI≥18 kg/m^2^. Exclusion criteria were: (1) end-stage renal disease; (2) current or previous history of malignancy; (3) pregnancy; and (4) use of anti-coagulant/anti-platelet medications. Recruited participants attended a one-off visit when written consent was obtained. Demographic data were collected including age, sex (based on hospital records) and ethnicity. Waist circumference and blood pressure were recorded, as well as medical and family history and current therapies. Retinopathy was determined by last retinal screening result (undertaken at least once per year). Healthy volunteers not on any treatment were recruited as control participants based on comparable age and sex characteristics to the study cohort, under institute ethics from the Leeds Institute of Cardiovascular and Metabolic Medicine (Medical Research Ethics Committee 19-006).

### eGDR

eGDR was calculated using the previously validated formula including waist circumference, HbA_1c_ and presence/absence of hypertension as follows: 21.158 − (0.09 × waist circumference [cm]) − (3.407 × hypertension [1=yes, 0=no]) − (0.551 × HbA_1c_ [%]) [17, 18].

### Venepuncture

Participants with type 1 diabetes and healthy control participants underwent standardised blood sampling with venepuncture conducted in a non-fasted state from the antecubital fossa using a 21G butterfly needle into citrate vacutainers [22]; the first drawn tube was discarded to minimise artefactual activation. Routine clinical tests in the type 1 diabetes group included HbA_1c_, liver function tests, lipid profile and full blood count.

### Multiparameter flow cytometry

A multiparameter flow cytometry panel was designed to allow simultaneous assessment of platelet activation, including: (1) conformational changes of integrin α_IIb_β_3_ [23], which facilitates the binding of fibrinogen and is measured through PAC-1-FITC (BD-Pharmigen 340507); (2) secretion of α-granules, measured through CD62P-R-phycoerythrin (PE) (BD-Pharmigen 555524); (3) phosphatidylserine (PS) exposure by analysing Annexin V (AnnV)-APC (BD Pharmigen 551061), which provides a haemostatic site for binding factor Xa [24, 25]; and (4) a platelet identification marker, CD42b-Brilliant Blue 700 (BB700) (BD Pharmigen 742219), as a component of the constitutively expressed glycoprotein Ib-IX-V (GPIb-IX-V) [26].

Samples were run on a CytoFLEX S (Beckman Coulter) using four detectors (525/40 BP, 585/42 BP, 660/10 BP and 712/25 BP) with CytExpert (v2.4) (Beckman Coulter) used to process all data. An automatic compensation was performed with VersaComp antibody capture beads and CytExpert v2.4. For the activation panel, whole blood was diluted 1:9 in assay buffer to minimise aggregate formation, then incubated with agonists or inhibitors and antibodies for 20 min at 37°C before fixation with paraformaldehyde solution (0.9% v/v) [26–28]. As AnnV binding is calcium dependent, these experiments were carried out in Modified Tyrode’s buffer supplemented with calcium (1.8 mmol/l) [26]. To induce platelet activation, blood was treated with protease-activated receptor-1 peptide (SFLLRN) and glycoprotein (GP) VI agonist cross-linked collagen-related peptide (CRP-XL), either alone or in combination to ensure a variety of stimulation strengths (low, medium and high) based on previously published data [26]. For inhibition experiments, whole blood was pre-incubated with PGI_2_ for 2 min before the addition of agonists. Platelets were gated on SSC/CD42b, excluding debris and doublets, and 10,000 CD42b^+^ events were recorded. A negative control of IgG-PE was included for CD62P-PE along with EDTA as the negative control for both PAC-1-FITC and AnnV-APC, as EDTA blocks calcium binding which is required both for integrin α_IIb_β_3_ conformational change and for AnnV binding. Gates were set on the matched isotype control and/or internal negative controls (EDTA) [27–29].

### FAUST

Full Annotation Shape-constrained Trees (FAUST) was used for the analysis of platelet subpopulations. This is a machine learning method that discovers and annotates cellular subpopulations within high-dimensional flow cytometry data [30]. This method annotates subpopulations on a per-sample basis. FAUST (v0.1.4; Tercen.com) was applied to the flow cytometric data from four individuals with high eGDR (normal insulin sensitivity) and three with low eGDR (insulin resistant). These were chosen to represent both ends of the spectrum of insulin resistance within the type 1 diabetes cohort. CD42b^+^ platelets were exported into FlowJo v10.10 (https://flowjo.com/) for data processing as previously described [31].

### Statistical analysis

CytExpert v2.4 was used to analyse all flow cytometry data. Statistical analysis was conducted using GraphPad Prism v10.1.1 (https://www.graphpad.com/). Statistical significance was determined as *p≤0.05, **p≤0.01, ***p≤0.001. For descriptive data, results are presented as median (interquartile range), mean ± SD and/or number (% of total). Normal distribution was assessed using Shapiro–Wilk test. One-way ANOVA (Kruskal–Wallis) was used to test multiple continuous variables. For the comparison of two independent continuous variables, either unpaired *t* test or Mann–Whitney was used, depending on the normative distribution of the data.

Power calculations were conducted based on SD derived from preliminary data and previously published results [27]. For CD62P mean fluorescence intensity (MFI) of expression following stimulation, based on an SD of 4000, a total sample size of 36 individuals would be sufficient to detect a difference of 4500 in MFI for this variable with a power of >80% at *p*<0.05, comparing those with and without insulin resistance*.* For PAC-1 binding, a total for 32 individuals would be required to detect a difference of 1500 in MFI, while 30 individuals were required to detect a difference of 1700 in MFI for AnnV binding, based on an SD for these variables of 1450 and 1770, respectively.

## Results

### Study participant characteristics

The median age of 32 study participants was 24 (range 18–34) years (59% male) with a mean ± SD duration of type 1 diabetes of 14.0 ± 6.3 years. Participants had a mean HbA_1c_ of 65.3 ± 14.0 mmol/mol (8.1%) with a mean eGDR of 8.1 ± 2.1 mg kg^−1^ min^−1^. No participants had macrovascular complications and just two were on additional non-insulin therapies (both metformin) (Table 1).

**Table 1:** Summary of the baseline characteristics of all study individuals with type 1 diabetes

Characteristic	Total
Number of participants	32
Male sex	19 (59)
Age, years	24.0 ± 3.8
Duration of diabetes, years	14.0 ± 6.3
HbA_1c_, mmol/mol	65.3 ± 14.0
HbA_1c_, %	8.1 ± 3.0
BMI, kg/m^2^	27.6 ± 5.6
eGDR (WC), mg kg^−1^ min^−1^	8.1 ± 2.1
Total daily insulin, U/24 h	66.0 ± 25.7
Total daily insulin, U/kg	0.8 ± 0.3
Total cholesterol:HDL ratio	3.2 ± 0.8
LDL-cholesterol, mmol/l	2.4 ± 0.8
Family history of T2D	4 (13)
Presence of retinopathy	13 (41)
Adjunctive therapy (metformin)	2 (6)
Macrovascular complications	0

Data are shown as mean ± SD or as *n* (%)

HbA_1c_ is given as DCCT, %

T2D, type 2 diabetes mellitus; WC, waist circumference

When stratifying participants according to their eGDR (Table 2), there were no statistically significant differences between groups in relation to sex, age, duration of diabetes or presence of retinopathy.

**Table 2:** Summary of the baseline characteristics of all study individuals with type 1 diabetes stratified according to eGDR

Characteristic	eGDR<6	eGDR 6–8	eGDR>8	*p* value
Number of participants	8	8	16	
Male sex	5 (63)	5 (63)	9 (56)	>0.1
Age, years	23.5 ± 3.5	23.2 ± 3.3	24.4 ± 3.2	>0.1
Duration of diabetes, years	11.2 ± 7.8	14.5 ± 5.1	14.8 ± 5.1	>0.1
HbA_1c_, mmol/mol	72.6 ± 9.8	74.4 ± 16.5	57.6 ± 16.6	0.028
HbA_1c_, %	8.8 ± 3.0	8.9 ± 4.0	7.4 ± 4.0	0.028
BMI, kg/m^2^	31.4 ± 8.4	28.7 ± 4.6	25.5 ± 4.6	>0.1
eGDR (WC), mg kg^−1^ min^−1^	5.1 ± 1.1	7.7 ± 0.6	9.7 ± 0.6	<0.0001
Total daily insulin, U/24 h	86.5 ± 22.7	72.8 ± 34.9	50.6 ± 34.9	0.024
Total daily insulin, U/kg	1.0 ± 0.2	0.9 ± 0.3	0.7 ± 0.34	0.058
Total cholesterol:HDL ratio	3.7 ± 0.5	3.4 ± 0.9	3.1 ± 0.6	>0.1
LDL-cholesterol, mmol/l	2.6 ± 0.8	2.2 ± 0.8	2.4 ± 0.8	>0.1
Family history of T2D	1 (13)	2 (25)	1 (6)	>0.1
Presence of retinopathy	2 (25)	4 (50)	8 (50)	>0.1
Adjunctive therapy (metformin)	2 (25)	0	0	>0.1
Macrovascular complications	0	0	0	>0.1

Data are shown as mean ± SD or as *n* (%)

HbA_1c_ is given as DCCT, %

T2D, type 2 diabetes mellitus; WC, waist circumference

### Platelet activation in type 1 diabetes and healthy control participants

**Fig. 1:**
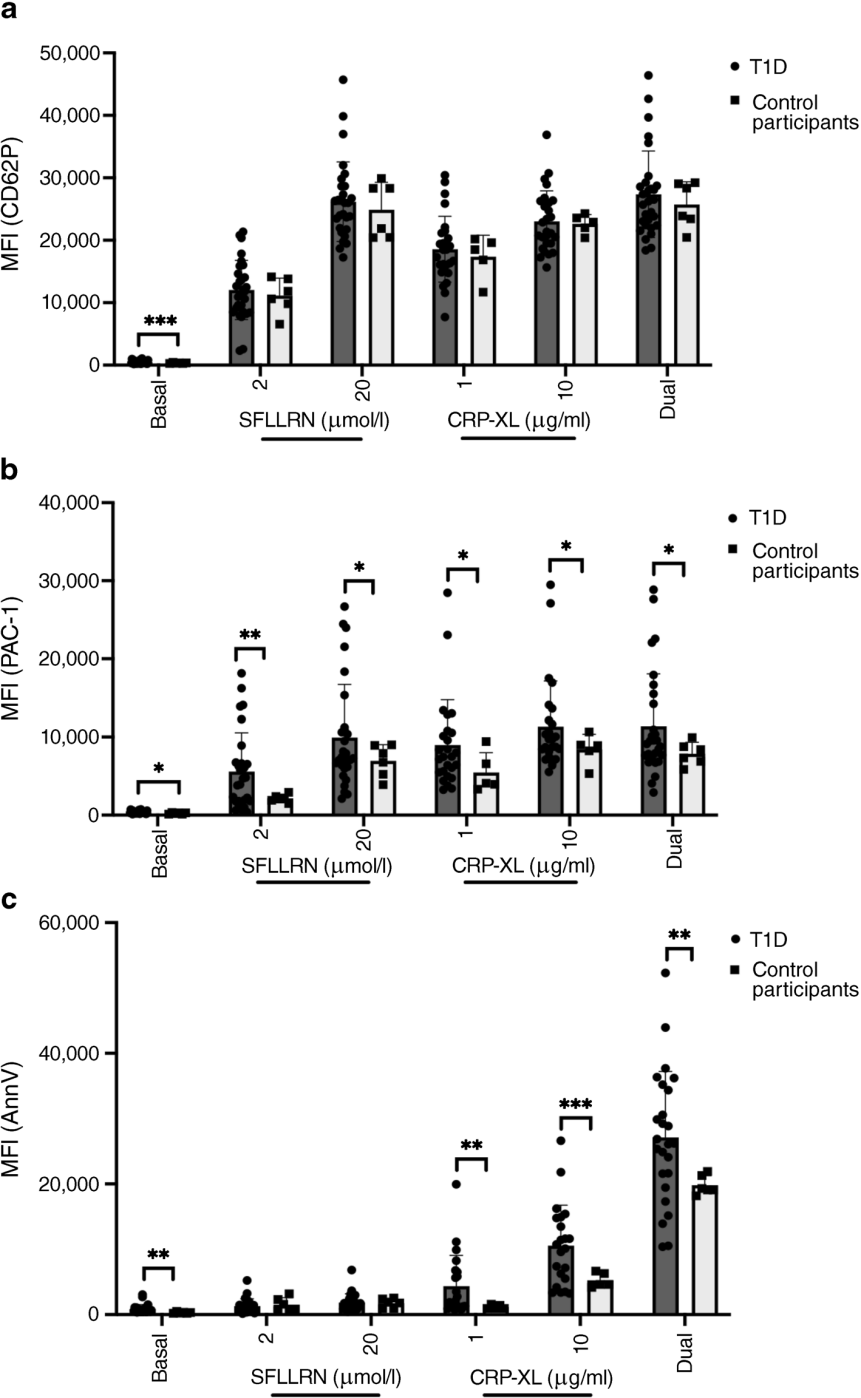
Multiparameter fluorescence flow cytometry to investigate platelet levels of (**a**) CD62P, (**b**) PAC-1 and (**c**) PS exposure in whole blood from study participants with type 1 diabetes (*n*=26–32) compared with healthy control participants (*n*=5–6). Expression is measured as MFI both at basal conditions and in response to stimulation with low and high dose single agonists (SFLLRN or CRP-XL) and high dose dual agonists (20 μmol/l SFLLRN and 10 μg/ml CRP-XL combined). *p≤0.05, **p≤0.01, ***p≤0.001. For comparison between two groups, unpaired *t* test or Mann–Whitney *U* tests have been used depending on distribution of data. Between multiple groups, ordinary ANOVA tests were carried out

Under basal conditions, platelets from individuals with type 1 diabetes expressed significantly greater levels of CD62P compared with control participants (521 ± 246 vs 335 ± 67; *p*<0.001), with similar findings for PAC-1 (370 ± 165 vs 231 ± 88; *p*=0.011) and PS (869 ± 762 vs 294 ± 109; *p*=0.001; Fig. 1a–c, electronic supplementary material [ESM] Fig. 1). Treatment of blood with either SFLLRN (a thrombin mimetic) or CRP-XL (a collagen mimetic) alone or in combination led to increased expression of all activation markers in both groups. Examination of CD62P showed no clear difference in expression in the type 1 diabetes group and control participants. In contrast, PAC-1 binding was elevated in the type 1 diabetes cohort compared with control participants when stimulated with single agonists (for example, at 2 μmol/l SFLLRN MFI was 5583 ± 4960 in type 1 diabetes vs 2155 ± 487 in healthy control participants, *p*=0.001 and at 10 μg/ml CRP-XL MFI was 11,335 ± 5124 vs 8419 ± 1942, *p*=0.03) or a combination of agonists (11,375 ± 6689 vs 7861 ± 1458, *p*=0.01), as shown in Fig. 1b. We also observed a greater propensity for PS exposure in type 1 diabetes platelets compared with healthy control participants following stimulation, with CRP-XL alone (at 1 μg/ml CRP-XL 4356 ± 4719 vs 1165 ± 308, *p*=0.004 and at 10 μg/ml CRP-XL 10,561 ± 6181 vs 5259 ± 1125, *p*<0.001) or in combination with SFLLLRN (27,124 ± 10,105 vs 19,801 ± 1455, *p*=0.001), which are known to induce PS exposure. Consistent with published studies, we found stimulation of the thrombin activation pathway alone is insufficient to stimulate PS [26, 27, 32]. Taken together, these data demonstrate that type 1 diabetes platelets are partially activated under basal conditions and that these same platelets exhibit a greater sensitivity to activation when challenged with agonists.

### Platelet activation in individuals with type 1 diabetes stratified by insulin resistance

Having observed platelet hyperactivity in individuals with type 1 diabetes compared with healthy control participants, we next examined the potential role of insulin resistance. The type 1 diabetes cohort was stratified according to eGDR, dividing into three groups: eGDR<6 mg kg^−1^ min^−1^, eGDR 6–8 mg kg^−1^ min^−1^ and eGDR>8 mg kg^−1^ min^−1^, to represent advanced, mild and normal insulin sensitivity, as per Nyström et al [18].

**Fig. 2:**
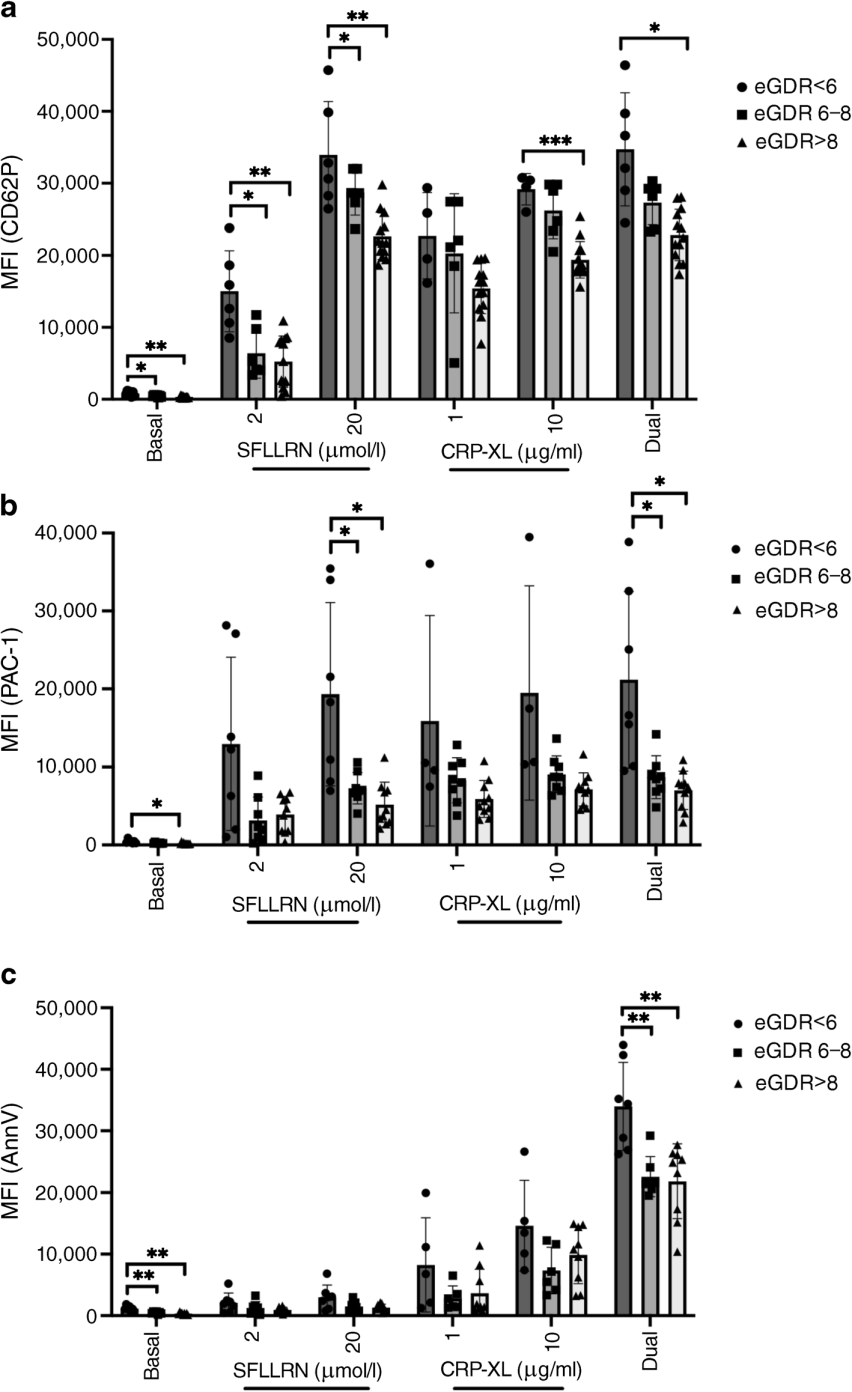
Multiparameter fluorescence flow cytometry to investigate platelet levels of (**a**) CD62P, (**b**) PAC-1 and (**c**) PS exposure in whole blood from study participants with type 1 diabetes (*n*=26–32) stratified according to eGDR threshold values derived from Nyström et al [18], eGDR<6 mg kg^−1^ min^−1^ (*n*=5–8), 6–8 mg kg^−1^ min^−1^ (*n*=6–9), >8 mg kg^−1^ min^−1^ (*n*=11–14). Expression is measured as MFI both at basal and in response to stimulation with low and high dose single agonists (SFLLRN or CRP-XL) and high dose dual agonists (20 μmol/l SFLLRN and 10 μg/ml CRP-XL combined). *p≤0.05, **p≤0.01, ***p≤0.001. For comparison between two groups, unpaired *t* test or Mann–Whitney *U* tests have been used depending on distribution of data. Between multiple groups, ordinary ANOVA tests were carried out

Under basal conditions, expression of all three activation markers was elevated in those with advanced insulin resistance compared with the other two groups (Fig. 2, ESM Fig. 2). When blood was treated with SFLLRN, CD62P was found to be elevated in individuals with advanced insulin resistance at 15,017 ± 5602 compared with mild insulin resistance (6304 ± 3478; *p*=0.01) or no insulin resistance (5226 ± 2565; *p*=0.007) (Fig. 2a). While we saw a similar pattern when examining PAC-1 binding, this only reached statistical significance at the higher concentration of SFLLRN, with advanced insulin resistance showing MFI of 19,339 ± 11,749 vs 7287 ± 2004 for mild insulin resistance (*p*=0.03; and 5187 ± 2872 for no insulin resistance [*p*=0.02], Fig. 2b). As expected, SFLLRN alone had no significant effect on PS (Fig. 2c).

To determine whether platelet hyperactivity in the advanced insulin resistance group was agonist-specific, we next tested the effect of CRP-XL. We observed elevated CD62P expression in the advanced insulin resistance group compared with those with normal insulin sensitivity in response to the higher concentration of CRP-XL (29,167 ± 2177 vs 22,829 ± 2535, *p*<0.001), while differences with PAC-1 and PS failed to reach statistical significance (Fig. 2b, c).

Using dual stimulation, those with advanced insulin resistance demonstrated elevated levels of CD62P, PAC-1 and PS compared with the other type 1 diabetes groups (Fig. 2).

### Platelet inhibition by PGI_2_ in individuals with type 1 diabetes and healthy control participants

Given previous work showing reduced platelet sensitivity to NO and PGI_2_ in type 2 diabetes [11], we speculated that platelet hyperactivity in type 1 diabetes may also be linked to disinhibition.

**Fig. 3:**
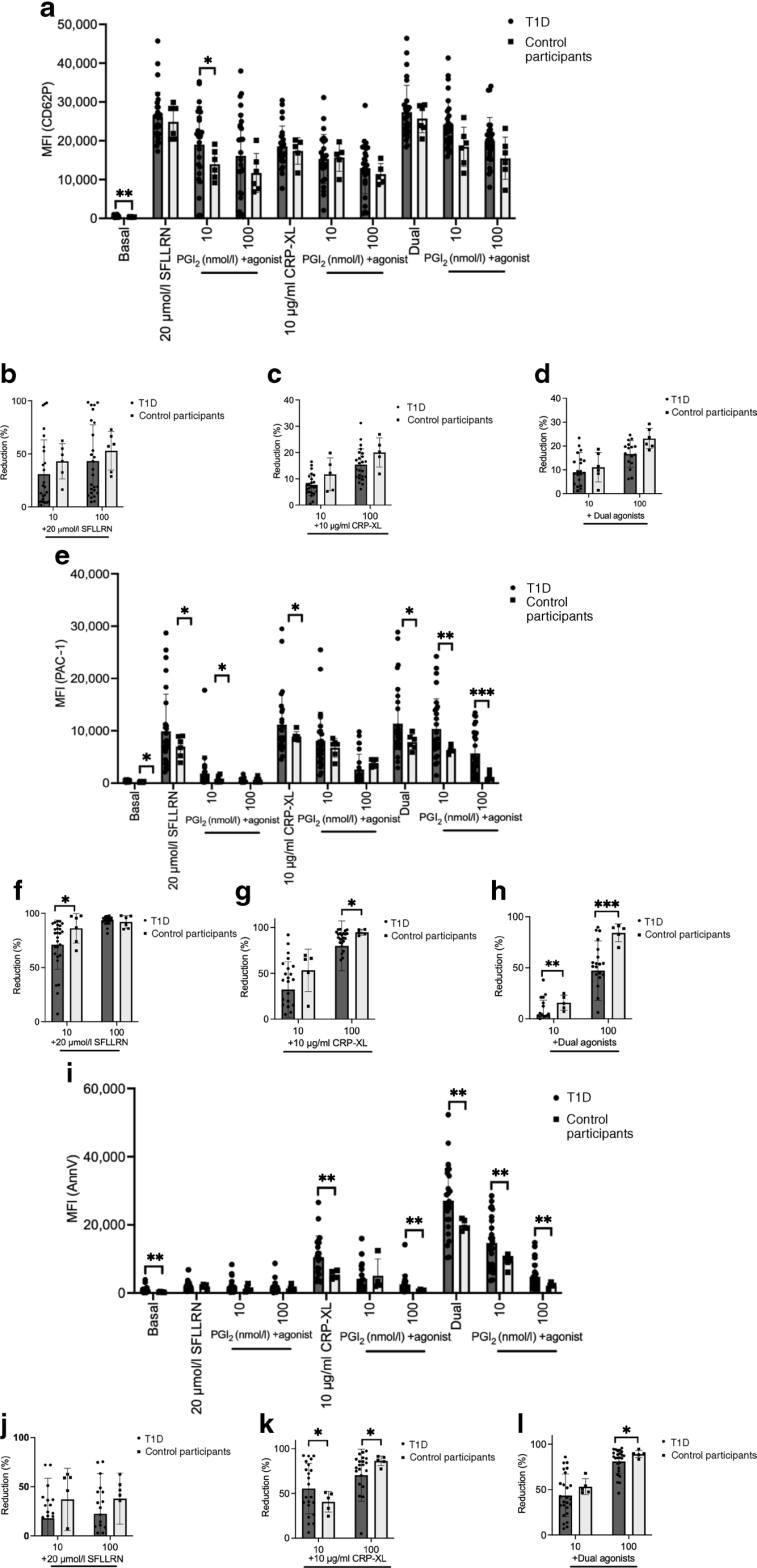
Multiparameter fluorescence flow cytometry to investigate platelet expression of (**a**) CD62P and (**e**) PAC-1 and (**i**) PS exposure in whole blood from study participants with type 1 diabetes (*n*=26–32) compared with healthy control participants (*n*=5–6). Expression is measured as MFI both at basal and in response to stimulation with high dose single agonists (SFLLRN or CRP-XL) and high dose dual agonists (20 μmol/l SFLLRN and 10 μg/ml CRP-XL combined) as well as in response to inhibition with PGI_2_ at low (10 nmol/l) and high (100 nmol/l) doses. (**b**–**d**, **f**–**h**,** j**–**l**) Response has been measured as percentage reduction compared with expression following stimulation at each of the agonist doses. *p≤0.05, **p≤0.01, ***p≤0.001. For comparison between two groups, unpaired *t* test or Mann–Whitney *U* tests have been used depending on distribution of data. Between multiple groups, ordinary ANOVA tests were carried out

There was no difference in sensitivity to PGI_2_ between type 1 diabetes and healthy control participants for CD62P expression (Fig. 3a–d). However, PAC-1 inhibition by the higher dose PGI_2_ was diminished in the type 1 diabetes group compared with control participants following stimulation with 10 μg/ml CRP-XL (reduction of 79 ± 18% vs 94 ± 3%, *p*=0.01) and the SFLLRN/CRP-XL combination at both inhibitor doses (3 ± 14% vs 16 ± 7%, *p*=0.007 at 10 nmol/l PGI_2_ and 47 ± 29% vs 84 ± 8%, *p*<0.001 at 100 nmol/l PGI_2_; Fig. 3h). When examining PS exposure, we again observed hyposensitivity to PGI_2_ inhibition in the type 1 diabetes group compared with control participants when platelets were stimulated with CRP-XL or a combination of SFLLRN/CRP-XL, which was only observed with the higher concentration of the inhibitor (Fig. 3i). Taken together, these data suggest hyposensitivity of type 1 diabetes platelets to the antithrombotic actions of PGI_2_.

### Platelet inhibition in individuals with type 1 diabetes stratified by insulin resistance

**Fig. 4:**
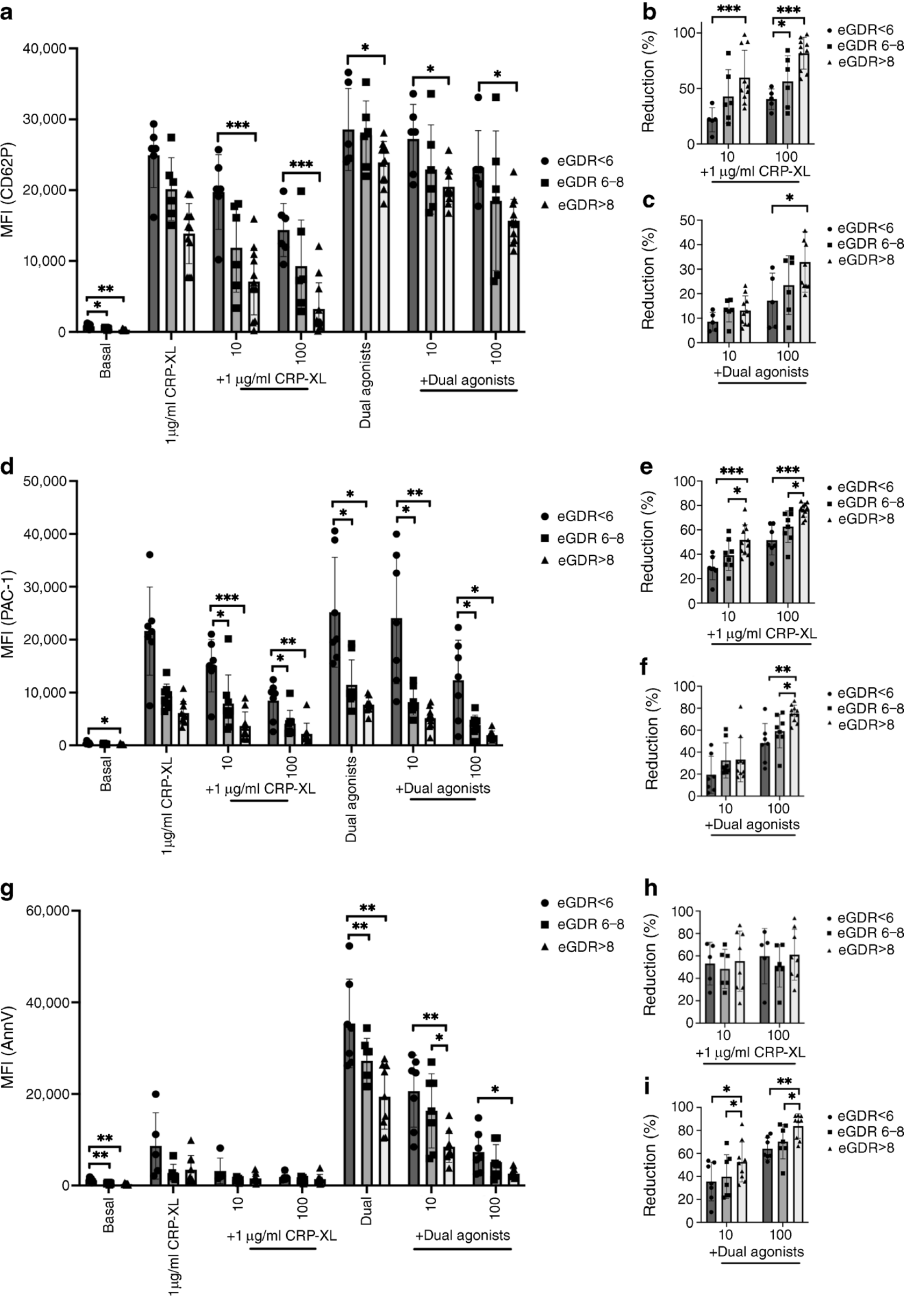
Multiparameter fluorescence flow cytometry to investigate platelet expression of (**a**) CD62P and (**d**) PAC-1 and (**g**) PS exposure in whole blood from study participants with type 1 diabetes (*n*=26–32), stratified according to eGDR threshold values derived from Nyström et al [18]. Expression is measured as MFI both at basal and in response to stimulation with single agonist (CRP-XL) and high dose dual agonists (20 μmol/l SFLLRN and 10 μg/ml CRP-XL combined) as well as in response to inhibition with PGI_2_ at low (10 nmol/l) and high (100 nmol/l) doses. (**b**, **c**, **e**, **f**, **h**, **i**) Response has been measured as percentage reduction compared with expression following stimulation at each of the agonist doses. *p≤0.05, **p≤0.01, ***p≤0.001. For comparison between two groups, unpaired *t* test or Mann–Whitney *U* tests have been used depending on distribution of data. Between multiple groups, ordinary ANOVA tests were carried out

Insulin resistance influenced platelet inhibition by PGI_2_, with less inhibition of CD62P observed in those with advanced insulin resistance (Fig. 4a) and with larger differences detected with PAC-1 and PS exposure (Fig. 4d–i). With 1μg/ml CRP-XL alone, the inhibition of PAC-1 by 10 nmol/l PGI_2_ in those with advanced insulin resistance was significantly reduced compared with those with normal insulin sensitivity (28 ± 9% vs 52 ± 2% *p*<0.001), which was also evident with higher PGI_2_ concentration (Fig. 4e). Following dual agonist stimulation, the higher concentration of PGI_2_ (100 nmol/l) showed diminished inhibition in the advanced insulin resistance group compared with the normal insulin sensitivity group (Fig. 4f). Similarly, the inhibition of PS exposure by low and high PGI_2_ concentrations was diminished in those with advanced insulin resistance compared with the other two groups (Fig. 4g–i). These data suggest that insulin resistance in type 1 diabetes is associated with a reduction in platelet sensitivity to the key endogenous inhibitor PGI_2_.

### Insulin resistance changes platelet subpopulation dynamics

The basis of functional platelet heterogeneity may lay in distinct receptor expression in response to physiological or pathophysiological mediators, along with size and sensitivity to activation [27]. Little is known regarding platelet subpopulations in individuals with type 1 diabetes. To address this, FAUST [31] was applied to flow cytometry data from unstimulated and dual agonist-stimulated platelets in the absence and presence of PGI_2_ from individuals with advanced insulin resistance or normal insulin sensitivity.

**Fig. 5:**
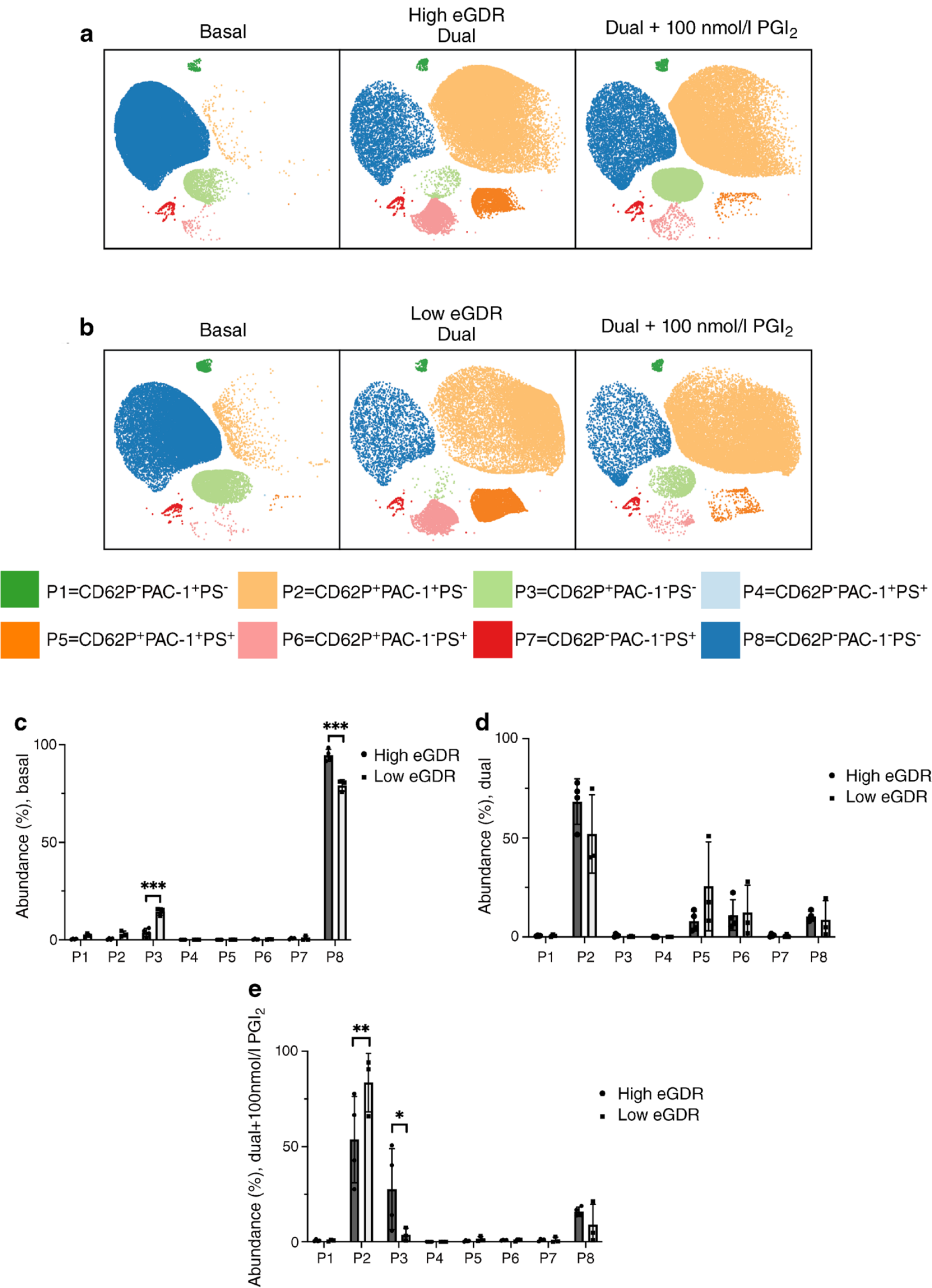
Platelet subpopulations. Whole blood was unstimulated (basal) or stimulated with SFLLRN and CRP-XL in the presence or absence of PGI_2_ (100 nmol/l) for 20 min prior to fixation. Samples were then analysed by flow cytometry where CD62P and PAC-1 expression and PS exposure were quantified. These data underwent platelet subpopulation analysis using FAUST. This discovered eight (P1–P8) platelet subpopulations present at basal, upon activation with SFLLRN and CRP-XL and in the presence of PGI_2_. These subpopulations were defined by differential CD62P, PAC-1 and AnnV binding. Platelet subpopulations are visualised on uniform manifold approximation and projection (UMAP) graphs from participants with (**a**) high eGDR (*n*=4) and (**b**) low eGDR (*n*=3). (**c**–**e**) Difference in platelet subpopulation abundance, presented in percentage (subpopulations, P1–P8), at basal (**c**), when dual agonist stimulated (**d**) and when dual agonist stimulated in presence of higher dose inhibition (100 nmol/l PGI_2_) (**e**). Data are expressed as mean ± SD. *p≤0.05, **p≤0.01, ***p≤0.001. For comparison between two groups, unpaired *t* test or Mann–Whitney *U* tests have been used depending on distribution of data. Between multiple groups, ordinary ANOVA tests were carried out

A total of eight platelet subpopulations (P1–P8) were detected in both cohorts, but critically with differing distribution (Fig. 5). These subpopulations were characterised by differential levels of CD62P, PAC-1 and PS, and they consisted of platelets with only activated α_IIb_β_3_, CD62P^−^PAC-1^+^PS^−^ (P1); platelets with α-granule secretion and activated α_IIb_β_3_, CD62P^+^PAC-1^+^PS^−^ (P2); platelets with only α-granule secretion, CD62P^+^PAC-1^−^PS^−^ (P3); platelets with activated α_IIb_β_3_ and PS exposure, CD62P^−^PAC-1^+^PS^+^ (P4); platelets with α-granule secretion, activated α_IIb_β_3_ and PS exposure, CD62P^+^PAC-1^+^PS^+^ (P5); platelets with α-granule secretion and PS exposure, CD62P^+^PAC-1^−^PS^+^ (P6); platelets with only PS exposure, CD62P^−^PAC-1^−^PS^+^ (P7); and resting platelets, CD62P^−^PAC-1^−^PS^−^ (P8).

Under basal conditions, the most abundant subpopulation was P8 for both normal insulin sensitivity and advanced insulin resistance groups (94.6 ± 1.5 and 79.2 ± 1.7, respectively), showing that most platelets in the total population are quiescent. However, P8 abundance was significantly lower in those with advanced insulin resistance (*p*<0.001), accompanied by a significant increase in P3 abundance (*p*<0.001). These data suggest that individuals with type 1 diabetes and advanced insulin resistance have a specific subset of activated circulating platelets under basal conditions, expressing elevated CD62P. Dual stimulation of platelets led to a remodelling of platelet subsets, with platelets moving from P8 to P2 (CD62P^+^PAC-1^+^PS^−^), P5 (CD62P^+^PAC-1^+^PS^+^) and P6 (CD62P^+^PAC-1^−^PS^+^), but we found no significant differences between groups. After treatment with PGI_2_, participants with advanced insulin resistance demonstrated a different inhibitory profile. Participants with advanced insulin resistance had significantly higher levels of P2 platelets (*p*=0.03) and significantly lower levels of P3 (*p*=0.006) when compared with those with normal insulin sensitivity. This suggests that those with advanced insulin resistance have increased CD62P expression and α_IIb_β_3_ activation in the presence of PGI_2_, implying that PGI_2_ is less effective at inhibiting the activation of these markers in individuals with advanced insulin resistance.

## Discussion

Individuals with type 1 diabetes are at increased risk of premature cardiovascular disease and different mechanisms have been proposed, including a prothrombotic environment [33]. Therefore, we explored platelet reactivity in type 1 diabetes as a composite of sensitivity to both platelet activators and inhibitors, while also determining the effects of insulin resistance in this population, which has never been studied before. We investigated younger adults with type 1 diabetes, to minimise the role of confounders, and employed multiparameter flow cytometry to understand platelet responses.

Examining the whole type 1 diabetes group, our first key observation was evidence of basal platelet activation. Previous studies have demonstrated increased CD62P expression, linked to increased platelet–neutrophil aggregates in type 1 diabetes [8, 34]; however, no differences in activated integrin α_IIb_β_3_ have been previously shown [35]. Our data are consistent with some of these findings, but we significantly expand our understanding by documenting that some platelets have activated integrin α_IIb_β_3_ and exposed PS at their surface under basal conditions. The elevated PS exposure likely accounts for an earlier observation demonstrating that type 1 diabetes platelets have elevated prothrombinase activity [36]. These data suggest that in vivo type 1 diabetes platelets are exposed to agents that induce low levels of activation, often referred to as priming agents, which reduce the threshold for activation and thrombosis [37, 38]. The identity of these priming agents is unknown, but examples could include oxidised LDLs (oxLDLs), advanced glycation end-products, plasma microparticles and insulin growth factor 1, all of which are elevated in type 1 diabetes [39, 40]. Consistent with platelet priming, we observed that platelets from type 1 diabetes were sensitised to further activation when exposed to agonists, consistent with a previous observation of hypersensitivity to thromboxane [41].

We next examined whether platelet modulation by PGI_2_ was compromised in type 1 diabetes. While PGI_2_ inhibited agonist-induced increases in platelet CD62P, integrin α_IIb_β_3_ and PS, the inhibitory effect of PGI_2_ was diminished in those with type 1 diabetes. We did not observe significant differences in CD62P inhibition, likely related to the reduced sensitivity of this platelet marker to PGI_2_, as we have previously documented [27]. Taken together, our data suggest that type 1 diabetes platelets display a maladaptive phenotype that is characterised by both agonist hypersensitivity and antagonist hyposensitivity. Importantly, all these platelet abnormalities are evident in these younger individuals and well before the development of clinical vascular complications.

Insulin resistance has been previously linked to platelet activation in type 2 diabetes [16], and we demonstrate it is a key determinant of abnormal platelet function in the setting of type 1 diabetes. In addition to increased basal platelet activation and a greater sensitivity to stimulation, platelets from type 1 diabetes with insulin resistance have diminished capacity for PGI_2_-mediated inhibition. Thus, while type 1 diabetes platelets generally appear to have abnormal activity, an insulin-resistant environment further amplifies platelet hyperreactivity. It can be argued that hyperglycaemia rather than insulin resistance contributed to these findings, given that HbA_1c_ is part of the eGDR calculation, but there were differences between individuals with advanced insulin resistance and those with intermediate insulin resistance, despite almost identical HbA_1c_ in these two groups, arguing against the findings being solely related to glycaemia.

To further understand the importance of this type 1 diabetes phenotype, we employed a machine learning tool to assess platelet subpopulations. It has been speculated that potent platelet activation leads to the generation of multiple subpopulations, characterised by surface marker expression, that may enact distinct functional roles [27]. This approach clearly showed that under basal conditions, individuals with type 1 diabetes had a population enriched in CD62P (P3; CD62P^+^PAC-1^−^PS^−^), which was absent in those with normal insulin sensitivity. Stimulation remodelled these populations such that the advanced insulin resistance group had a higher subpopulation of fully activated platelets (P5; CD62P^+^PAC-1^+^PS^+^). Despite incubation with the most potent physiological platelet inhibitor, platelets from individuals with insulin resistance continued to express CD62P and activated integrin α_IIb_β_3_, making them primed to participate in platelet–neutrophil and platelet–fibrinogen interactions, respectively. Individuals with a greater proportion of pro-aggregatory (CD62P^+^PAC-1^+^) subpopulations may benefit from proactive anti-platelet therapy. Additionally, our data and previously published work support that CD62P has diminished sensitivity to PGI_2_ inhibition and therefore individuals with high CD62P^+^ may benefit from targeting alternative pathways of inhibition.

Although the markers described would not be measured in clinical practice, this work has important future management implications. Individuals with type 1 diabetes are largely treated as one homogeneous cohort, but our data suggest this ‘one size fits all’ approach may not be adequate and highlight the need for individualised care. Incorporating eGDR calculations, using easily available clinical parameters, should help to further assess thrombotic risk and tailor future therapies accordingly. Moreover, individuals with a greater proportion of pro-aggregatory (CD62P^+^PAC-1^+^) subpopulations may benefit from proactive anti-platelet therapy, particularly when deranged PGI_2_ inhibition is evident.

Understanding platelet subpopulations in individuals with type 1 diabetes will help to establish different thrombotic phenotypes, consequently facilitating future person-specific antithrombotic therapies in those with clinically relevant vascular disease. Our data demonstrate heterogeneity within the platelet population with potential for distinct functional roles, e.g. pro-aggregatory vs pro-coagulant platelet sub-types, and, therefore, an individualised therapeutic approach may be beneficial. However, the clinical translation of our findings will require future research using a combination of in vivo and ex vivo work to understand the factors that restore normal platelet physiology and reduce the risk of thrombosis in the insulin-resistant type 1 diabetes population.

There are a number of strengths to this study that should be highlighted. This is the first piece of work to characterise PS exposure specifically in type 1 diabetes and also to demonstrate elevated integrin α_IIb_β_3_. Furthermore, it is also the first study to fully explore response to inhibition using multiparameter flow cytometry in type 1 diabetes, allowing simultaneous investigation of different aspects of platelet activation. In addition, this is the first time the impact of insulin resistance on platelet activation and inhibition in type 1 diabetes has been investigated.

Equally, there are limitations that must be considered. A limited number of comparable healthy control participants based on age and sex were included in the initial experiments. The study was designed to first establish whether individuals with type 1 diabetes had evidence of increased platelet hyperreactivity compared with healthy control participants to then allow greater focus on the role of insulin resistance within the cohort of individuals with type 1 diabetes. As the results showed statistical difference and based on the described power calculations, these preliminary results were adequately powered and the numbers included thus reflect this. Next, it could appear that the results may be related to glycaemia rather than insulin resistance as mentioned. However, as outlined above, glycaemia was not the sole determinant of platelet propensity to activation and resistance to inhibition. Specific analysis by sex was not performed due to the relatively small cohort. Future studies with a greater number of participants could explore any sex-specific differences. The study was also limited to young adults (aged 18–40), but given that mean diabetes duration was 14 years, it is likely these findings are applicable to individuals living with type 1 diabetes across all ages. The exact contribution of these platelet abnormalities to future cardiovascular risk in this cohort is currently unknown and remains an area for future research.

Future work focusing on more detailed mechanistic aspects may provide crucial insight and identify potential therapeutic targets for the future. While our experiments focused on two widely used platelet agonists (CRP-XL and SFLLRN), further experiments with other agonists such as oxLDL that have close relevance to individuals with insulin resistance would be another interesting aspect of future work. Further work may also include exploring the kinetics of platelet activation which may have therapeutic implications [42].

In conclusion, we present a novel characterisation of platelets in younger adults with type 1 diabetes that shows increased activation under basal conditions, overactivation following stimulation and a diminished response to inhibition. Insulin resistance appears to further drive this phenotype, suggesting that those with a combination of type 1 diabetes and insulin resistance are at even greater platelet-driven cardiovascular risk. Clinically, this would support the need for more widespread identification of insulin resistance in the type 1 diabetes population and an individualised approach to both insulin-sensitising adjunctive and antithrombotic therapies. Future research is needed to understand whether amelioration of insulin resistance in type 1 diabetes improves this maladaptive platelet phenotype and whether more aggressive antithrombotic strategies in those with insulin resistance alter long-term vascular outcomes.

## Supplementary Information

Below is the link to the electronic supplementary material.

ESM Figures (PDF 158 KB)

## Data Availability

Data will be provided by the corresponding author via email upon reasonable request

## Abbreviations

AnnV: Annexin V
CRP-XL: Cross-linked collagen-related peptide
eGDR: Estimated glucose disposal rate
FAUST: Full Annotation Shape-constrained Trees
MFI: Mean fluorescence intensity
NO: Nitric oxide
oxLDL: Oxidised LDL
PE: R-phycoerythrin
PGI_2_: Prostacyclin
P1–P8: Platelet subpopulations 1–8
PS: Phosphatidylserine
