# Supplementary material for: Insulin resistance in type 1 diabetes is a key modulator of platelet hyperreactivity
Source: Diabetologia. 2025 Apr 30;68(7):1544–58. doi: 10.1007/s00125-025-06429-z (PMC12176951; doi:10.1007/s00125-025-06429-z)
Supplement: Supplementary file 1 — ESM Figures (PDF 158 KB) [file 125_2025_6429_MOESM1_ESM.pdf]

Electronic supplementary data.

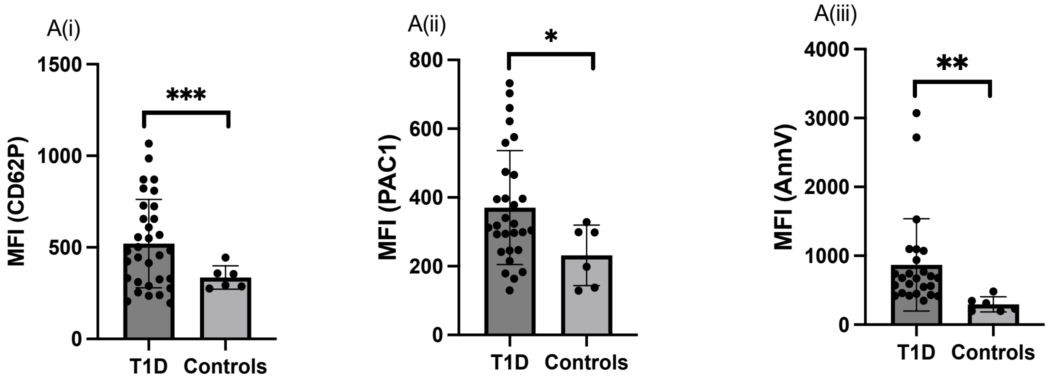

ESM Figure 1; Multiparameter fluorescent flow cytometry to show platelet expression under basal conditions for patients with T1D and healthy controls for A(i) CD62P, A(ii) PAC1 and A(iii) PS. **p<0.05, ** p<0.01, ***p<0.001.*

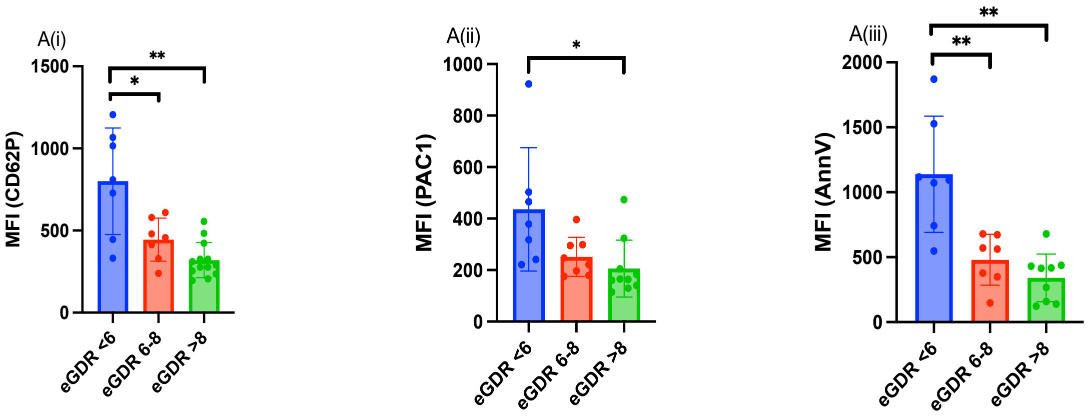

ESM Figure 2; Multiparameter fluorescent flow cytometry to show platelet expression under basal conditions for patients with T1D stratified according to insulin resistance measured by eGDR. Markers investigated are; A(i) CD62P, A(ii) PAC1 and A(iii) PS. **p<0.05, ** p<0.01, ***p<0.001.*
